# A Latin American Man with Palpitations, Dizziness, Episodes of Nonsustained Ventricular Tachycardia, and an Apical Aneurysm

**DOI:** 10.1371/journal.pntd.0000852

**Published:** 2011-02-22

**Authors:** Anis Rassi, Anis Rassi, Carlos Franco-Paredes

**Affiliations:** 1 Section of Cardiology, Anis Rassi Hospital, Goiânia, Brazil; 2 Department of Medicine, Emory University School of Medicine, Atlanta, Georgia, United States of America; Weill Medical College of Cornell University, United States of America

## Case Description

**Figure 1:**
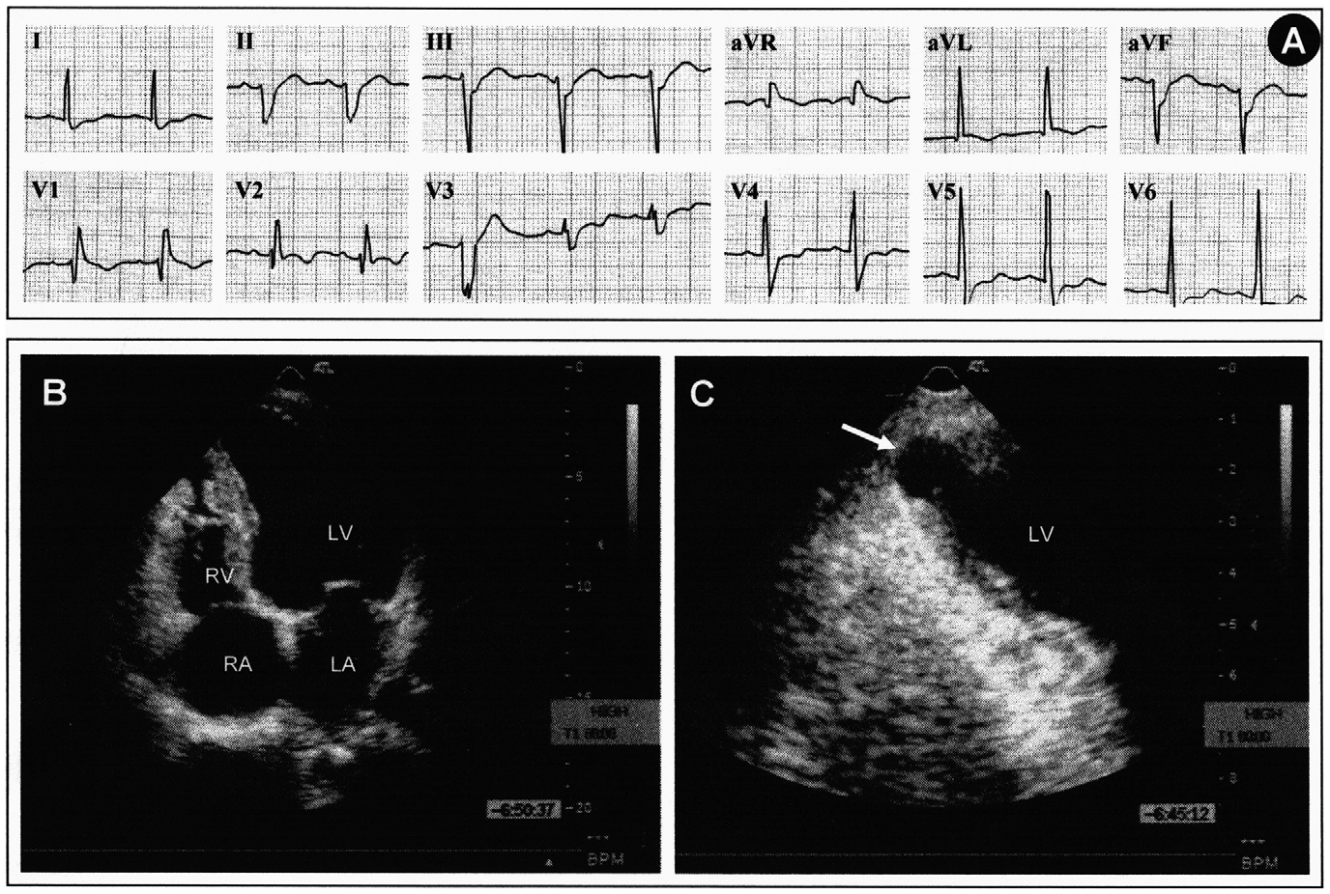
Patient characteristics. (A) 12-lead ECG showing the three most frequent abnormalities in Chagas heart disease: right bundle branch block, left anterior fascicular block, and a premature ventricular contraction (lead V3). (B) Transthoracic echocardiogram recorded at the four chamber apical view showing slightly diminished left ventricular systolic function and mildly dilated left ventricle. (C) Two chamber apical view showing more clearly a characteristic digitiform left ventricular apical aneurysm (arrow).

**Figure 2:**
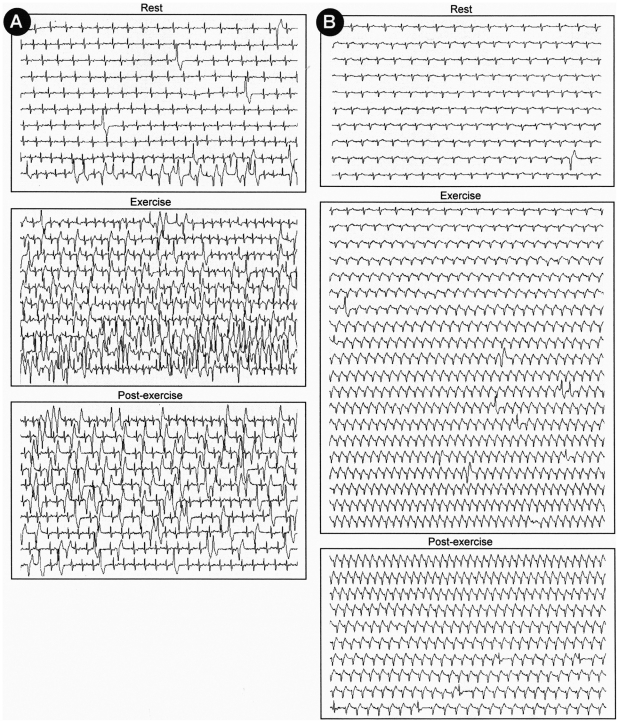
Exercise testing performed and recorded during the last hour of Holter monitoring. (A) Control exercise showing frequent premature ventricular complexes, couplets, and short runs of nonsustained ventricular tachycardia. (B) Exercise testing after administration of amiodarone (400 mg/day), showing a significant reduction in the number of premature ventricular complexes and couplets, and a complete abolition of episodes of nonsustained ventricular tachycardia.

A 43-year-old man from a rural area in Brazil presented to his physician with complaints of palpitations and lightheadedness, mainly on exertion. An electrocardiogram (ECG) showed right bundle-branch block (RBBB), left anterior fascicular block (LAFB), and a premature ventricular contraction (PVC) (Figure 1A). Echocardiography revealed a mildly depressed left ventricular (LV) ejection fraction (EF) of 46%, a slightly increased LV end-diastolic diameter (60 mm), and a LV apical aneurysm (Figure 1B and 1C). Subsequent 24-hour ambulatory ECG monitoring (Holter) recorded 4,055 isolated polymorphic PVCs, 236 couplets, and 17 short runs of nonsustained ventricular tachycardia (NSVT). Exercise testing revealed several episodes of NSVT (Figure 2A). The patient was initially treated with an angiotensin-converting enzyme (ACE) inhibitor and amiodarone (loading dose of 600 mg/day for 1 week, followed by 300 mg/day). One month later, Holter and exercise testing were repeated, showing a significant reduction in total PVCs on Holter, but with persistence of few episodes of NSVT on exercise testing. The dose of amiodarone was increased to 400 mg/day, both tests were repeated, and abolition of NSVT was then obtained (Figure 2B).

## Diagnosis

**Chagas heart disease (chronic chagasic cardiomyopathy).** Chagas heart disease (CHD) is the most common etiology of cardiomyopathy in Latin America, and a major cause of cardiovascular death among middle-aged individuals in endemic areas. CHD is also the most serious and frequent manifestation of chronic Chagas disease. It develops in 20%–30% of infected persons, usually 10–30 years after *Trypanosoma cruzi* contamination, and manifests as three major syndromes that often coexist in the same patient: arrhythmias, heart failure, and thromboembolism [1].

Arrhythmias are very common, of different types, and may cause palpitations, dizziness, syncope, and sudden cardiac death (SCD). Frequent, complex PVCs, including couplets and runs of NSVT, are a common finding on 24-h Holter monitoring and exercise testing. They correlate with the severity of ventricular dysfunction, but can also occur in patients with quite well preserved ventricular function. Episodes of NSVT are seen in approximately 40% of patients with mild wall motion abnormalities and in virtually all patients with heart failure, an incidence that is higher than that observed in other cardiomyopathies [2].

Heart failure is often a late manifestation of CHD. It is usually biventricular with a predominance of left-sided failure at initial stages and of right-sided failure at more advanced disease. Systemic and pulmonary embolisms arising from mural thrombi in the cardiac chambers are quite frequent. Clinically, the brain is by far the most frequently recognized site of embolisms (followed by limbs and lungs), but at necropsy, embolisms are found more frequently in the lungs, kidneys, and spleen [3]. SCD is the main cause of death in patients with CHD, accounting for nearly two-thirds of all deaths, followed by refractory heart failure (25%–30%) and thromboembolism (10%–15%) [4].

Electrocardiographic alterations are varied, but the most common are RBBB, LAFB, PVCs, primary ST-T changes, Q waves, low QRS voltage, different degrees of atrioventricular block, and manifestations of sinus node dysfunction. All these alterations may be isolated or associated. The association of RBBB with LAFB strongly suggests chronic CHD in endemic regions [1], [5].

On the chest radiography, cardiac size is normal in the initial stages of disease and even when multiple ECG abnormalities are present. The cardiac size may be slightly, moderately, or severely increased at later stages of CHD. In nearly half of the cases with heart failure, the manifestations of pulmonary congestion are poor or even absent [1], [5]. The echodopplercardiogram may be abnormal even in patients with nonspecific ECG alterations and normal chest radiography. Echo shows wall motion abnormalities in two main areas of LV: the apex and the posterior-inferior wall. The most characteristic findings are apical aneurysms (with or without thrombi) [1], [5]. Three types of aneurysms have been described: digitiform (the most common), mammilar, and saccular. More advanced disease is characterized by global ventricular dilatation and diffuse hypokinesis, often associated with mitral and tricuspid regurgitation [1], [5].

Holter monitoring is an excellent method for investigating patients with CHD. It may be used to identify complex ventricular arrhythmias, diagnose transitory arrhythmias, and evaluate anti-arrhythmic therapy. The exercise testing evaluates the functional capacity of the patient, qualifies and quantifies PVCs, and may verify the efficacy of anti-arrhythmic drugs.

In patients with high suspicion of chronic Chagas disease or in those with a compatible clinical syndrome, because parasitaemia is scarce, the presence of immunoglobulin G (IgG) antibodies against *T. cruzi* antigens needs to be detected by at least two different serological methods (usually enzyme-linked immunosorbent assay, indirect immunofluorescence, or indirect haemagglutination) to confirm the etiological diagnosis.

The initial evaluation of the newly diagnosed patient with chronic *T. cruzi* infection includes a complete medical history and physical examination, and a resting ECG [1], [5], [6]. Patients with ECG changes consistent with CHD should undergo a routine cardiac evaluation, including ambulatory 24-h Holter monitoring (complemented with an exercise testing whenever possible) to detect arrhythmias and assess functional capacity; chest radiography and echocardiography to refine the assessment of cardiac size and function, and to incorporate additional prognostic information; and other cardiologic tests as indicated. From the results of these tests, clinicians can stratify individual patients by risk and implement appropriate treatment [1], [5], [6].

Recently, some of us used a rigorous multivariate analysis to develop a risk score for mortality prediction in 424 outpatients with CHD from a regional Brazilian cohort [7]. Several demographic, clinical, and noninvasive variables were tested, and six were identified as independent predictors of mortality and were assigned points according to the strength of their statistical association with the outcome: New York Heart Association (NYHA) class III or IV (5 points), cardiomegaly (5 points), LV systolic dysfunction on echocardiography (3 points), NSVT on Holter (3 points), low QRS voltage (2 points), and male gender (2 points). From addition of the points to provide the risk score, patients were classified into groups of low (0–6 points), intermediate (7–11 points), and high risk (12–20 points), with corresponding 10-year mortality rates of 10%, 44%, and 84%, respectively [7].

The clinical history, the ECG abnormalities, and the findings on echo, Holter, and exercise testing in the present case are characteristic of chronic CHD. Serological tests for Chagas disease were performed and gave positive results. According to the scoring system to determine the risk of death described above, this patient would receive a score of 8 points based on his sex (2 points), the presence of apical aneurysm (3 points), and the presence of runs of NSVT (3 points). This score puts him in the intermediate-risk category (risk of death in the next 10 years of 44%).

The importance of having the results of a chest radiography on board, which was not done in this case, relies on the fact that the presence of cardiomegaly would add 5 more points to the patient's risk score, moving him to a higher-risk category. Of note, the combination of NSVT and LV systolic dysfunction in patients with CHD increase the risk of subsequent death by 15 times compared to patients without these abnormalities on Holter monitoring and on echocardiography [8].

Management of patients with CHD should focus on eradication of the parasite, which may halt disease progression, alleviation of symptoms of disease, and identification of patients at high risk of death, in order to implement strategies with the potential to reduce mortality. Regarding antitrypanosomal treatment, only two agents are currently available: benznidazole and nifurtimox. On the basis of the results of some observational studies, a panel of experts convened by the US Centers for Disease Control and Prevention in 2006 recommended that antiparasitic therapy should generally be offered to adults 19–50 years of age with mild to moderate cardiomyopathy [6]. A multicenter randomized controlled trial is currently under way to assess whether trypanocidal therapy affects mortality and cardiovascular outcomes in 3,000 patients aged 18–75 years with chronic CHD without advanced cardiac involvement, but results will not be available until 2012 at the earliest [9]. Regarding antiarrhythmic therapy, despite a scarcity of data, individuals at high risk of SCD such as the patient in this vignette, are usually treated with amiodarone. Amiodarone markedly reduces the frequency and complexity of ventricular arrhythmias and has been shown to reduce mortality in the only two randomized trials that included chagasics patients (EPAMSA [10] and GESICA [11]). The efficacy of amiodarone was evaluated by a noninvasive approach, which uses repeated ambulatory monitoring and exercise testing [12]. Criteria for drug efficacy depend upon the suppression of spontaneously occurring arrhythmias. Support for the use of this approach is based on the observation that the presence of runs of NSVT significantly increases the risk of death. Moreover, their prevention with amiodarone may improve survival [12].

Because some evidence suggests that mild segmental LV wall motion abnormalities are predictors of deterioration of ventricular function during follow-up [13], the use of an ACE inhibitor is also highly recommended. Finally, whether administration of a betablocker (to prevent further myocardial damage and death) and either aspirin or warfarin (to prevent thromboembolism) should be considered for this patient remains an open and challenging question.

The ethics committee of Anis Rassi Hospital approved this report and waived the need for informed consent.

**Key Learning Points**

- Clinical manifestations of Chagas heart disease are diverse and related to three major syndromes: cardiac arrhythmias, heart failure, and thromboembolism. Improved models for risk stratification are now available, and certain guided treatments may increase survival.
- Segmental or global left ventricular dysfunction and episodes of nonsustained ventricular tachycardia are independent, additive risk factors for overall mortality in Chagas cardiomyopathy.
- Although not supported by incontestable evidence, it is possible that patients with runs of nonsustained ventricular tachycardia on Holter/exercise testing, and asymptomatic or mildly symptomatic left ventricular dysfunction on echocardiography, derive some benefit from combined therapy with an angiotensin-converting enzyme inhibitor and amiodarone.
- Persistence of parasites and the accompanying chronic inflammation is the basis for the pathology in Chagas heart disease. Thus, unless proved otherwise by a randomized controlled trial, benznidazole or nifurtimox should usually be offered to patients <50 years old with presumably long-standing indeterminate *T. cruzi* infections or even with mild to moderate disease.
- The potential benefits of novel therapies (e.g., betablockers, implantable cardioverter defibrillators) need assessment in prospective randomized trials. The best therapeutic strategy for patients with apical aneurysm not containing thrombus is unknown.
